# Nævus Maternus

**Published:** 1847-01

**Authors:** 


*Nævus Maternus.*—Here, gentlemen, we have an affection which is by no means uncommon. You hear the history of the case. The patient is a fine healthy child, 4 months old, with a congenital mark on the breast, which was quite small at birth, and has been gradually increasing till it has attained its present size. The cause of this disease is unknown; it has been referred to the mother's longing for, or aversion to cherries, strawberries, mulberries, grapes, etc., and on that account has received the name of *Nævus Maternus*, the mother's mark; as regards the probability of this theory, I leave you to judge. Now this affection is purely vascular. You know we have three varieties of Aneurism, first the Venous, second the Arterial, and third the Anterio-Venous, or the Aneurism by Anastomosis, forming a perfect net-work of vessels; the case before us is an example of the latter. These marks occur on all parts of the body, and often go on increasing, till they have attained a certain size, when they ulcerate or slough, thereby causing violent, and often fatal hemorrhage. Now there are various modes of treating these cases; one is to excise the tumor, which, I believe, is at the present day, abandoned; another to pass threads through the part, and allow them to remain there until inflammation is excited, which converts it into a hard tumor. Vaccination is a third method which has been resorted to, so performed as to involve the whole tumor. Applications of caustic to the part have also been made, causing it to slough away; and finally, the actual cautery is used, to which I give the preference. You can always promise a cure in these cases, and the treatment which I have found very successful, is to pass needles heated to whiteness in the flame of a spirit lamp, five or six times through the tumor; inflammation follows, and the tumor gradually sloughs away. It will be necessary sometimes, to repeat the operation, and in this manner you can very readily get rid of the difficulty. After the operation, the application of lint, satured with cold water, to the parts, will be found very serviceable.

